# Safety and Effectiveness of Repeated Botulinum Toxin A Intracavernosal Injections in Men with Erectile Dysfunction Unresponsive to Approved Pharmacological Treatments: Real-World Observational Data

**DOI:** 10.3390/toxins15060382

**Published:** 2023-06-05

**Authors:** François Giuliano, Pierre Denys, Charles Joussain

**Affiliations:** 1 Neuro-Uro-Andrology R. Poincare University Hospital, AP-HP, 104 Bvd R. Poincare, 92380 Garches, France; pierre.denys@aphp.fr (P.D.); charles.joussain@aphp.fr (C.J.); 2 UMR 1179, Inserm Faculty of Medicine, Versailles Saint Quentin University, Paris Saclay, 78180 Montigny le Bretonneux, France

**Keywords:** onabotulinumtoxinA, abobotulinumtoxinA, incobotulinumtoxinA phosphodiesterase type 5 inhibitors, prostaglandinE1

## Abstract

Intracavernosal injections of botulinum toxin A (BTX/A *ic*) may be effective for difficult-to-treat erectile dysfunction (ED). This is a retrospective case series study of the effectiveness of repeated off-label BTX/A *ic* (onabotulinumtoxinA 100U, incobotulinumtoxinA 100U or abobotulinumtoxinA 500U) in men with ED and insufficient response to phosphodiesterase type 5 inhibitors (PDE5-Is) or prostaglandinE1 intracavernosal injections (PGE1 ICIs), defined as an International Index of Erectile Function-Erectile Function domain score (IIEF-EF) < 26 on treatment. Further injections were performed on patients’ requests, and the files of men who underwent at least two injections were reviewed. The response to BTX/A *ic* was defined as the achievement of the minimally clinically important difference in IIEF-EF adjusted to the severity of ED on treatment at baseline. Out of 216 men treated with BTX/A *ic* and PDE5-Is or PGE1-ICIs, 92 (42.6%) requested at least a second injection. The median time since the preceding injection was 8.7 months. In total, 85, 44 and 23 men received, respectively, two, three and four BTX/A *ic*. The overall response rate was 77.5%: 85.7% in men with mild ED, 79% for moderate ED and 64.3% for severe ED on treatment. The response increased with repeated injections: 67.5%, 87.5% and 94.7%, respectively, after the second, third and fourth injections. Post-injection changes in IIEF-EF were similar across injections. The time from injection to request for a further injection varied little. Four men reported penile pain at the time of injection (1.5% of all injections), and one experienced a burn at the penile crus. Repeated BTX/A injections combined with PDE5-Is or PGE1-ICIs produced an effective and durable response, with acceptable safety.

## 1. Introduction

The pharmacological treatment for erectile dysfunction (ED) of any etiology is well standardized. The first-line treatment is oral phosphodiesterase type 5 inhibitors (PDE5-Is). If that fails, on-demand intracavernosal injections (ICIs) of vasoactive substances, such as prostaglandin E1 (PGE1) alone or in combination with papaverine (Bi-mix) and phentolamine (Tri-mix) [1], are performed. The rate of insufficient, or absence of, effectiveness of PDE5-Is is approximately 30% [2,3]. The failure rate is even higher in specific subgroups, e.g., when the etiology of ED is diabetes mellitus or radical prostatectomy [4,5]. Despite their greater effectiveness, the acceptability of ICIs is poor, particularly because of the lack of spontaneity of sexual activity when using this treatment [6]. Accordingly, alternative non-pharmacological therapeutic options for ED are currently under investigation. Low-intensity extracorporeal shock wave therapy (Li-ESWT) seems promising for mild vasculogenic ED and men with vasculogenic ED who are poorly responsive to PDE5-Is. However, the current level of evidence is still low, and the long-term effect remains unknown [7]. The level of evidence for stem cell therapy and platelet-rich plasma injections is still very low [8,9]. Intracavernosal botulinum toxin A (BTX/A *ic*) is another alternative pharmacological option that is currently emerging [10]. The results of randomized controlled clinical trials [11,12,13] and uncontrolled [14,15,16] studies of single BTX/A *ic* injections suggest the treatment is effective and has a good safety profile in men with difficult-to-treat ED. In those studies, 50 then 100U of onabotulinumtoxinA, 250 then 500 Speywood U of abobotulinumtoxinA and 100U of incoabotulinumtoxinA were found to be effective and to have acceptable tolerability. BTX/A injections have been approved for a variety of indications targeting conditions involving striated muscles, e.g., spasticity, wrinkles and blepharospasm or smooth muscle cells, e.g., neurogenic detrusor overactivity (NDO) or idiopathic overactive bladder (IOAB) [17]. BTX/A *ic* is believed to facilitate cavernosal smooth muscle relaxation and penile arterial vasodilation [18]. Intradetrusor BTX/A injections for the treatment of NDO or IOAB must be repeated every 6 to 8 months because of a progressive decrease in the therapeutic effect [19]. It is currently unknown if this requirement is similar for the treatment of ED using BTX/A *ic*. 

The aim of this study was to determine the frequency at which BTX/A *ic* needed to be repeated and the effectiveness of repeated injections on erectile function in men with difficult-to-treat ED using real-life data from men treated with repeated BTX/A *ic* in our department in the past few years. We also wished to examine the consistency of the treatment response and the safety of the procedure.

## 2. Results

**Figure 1:**
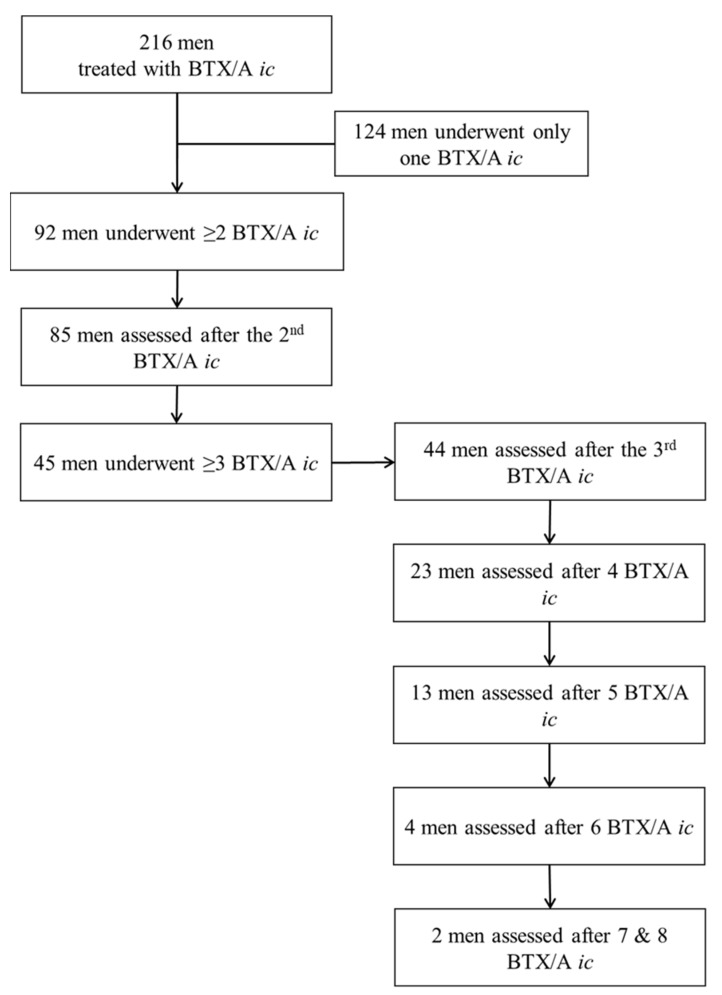
Flowchart of men treated with BTX/A *ic*.

Between July 2017 and November 2022, we treated 216 men with BTX/A *ic* in addition to their existing pharmacological treatment. Of these, 92 (42.6%) requested at least a second injection with a median delay of 8.95 (7.5–13.8) months since the first. The baseline characteristics of these participants are shown in Table 1. The study was conducted in a tertiary center specializing in the management of uro-genito-sexual dysfunctions in people with neurologic disorders, which explains the overrepresentation of men with spinal cord injury in the series. Figure 1 shows the study flowchart. 

**Figure 2:**
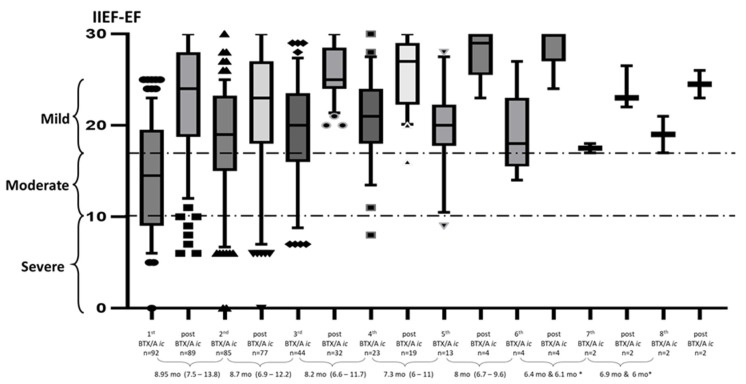
Box and whisker plots (10^th^–90^th^ percentile) of the Erectile Function domain score of the International Index of Erectile Function (IIEF-EF) at the time of each repeated BoNT/A intracavernosal injection (BTX/A *ic*) and during the second month (mo) post-injection. * the median was not calculated as only two men underwent injections.

The median time from previous injection to request for a further injection (second, third or fourth) was 8.7 (6.9; 12.2) months. Figure 2 shows the median changes in IIEF-EF domain score across each injection repetition in the overall cohort. Post-injection changes in IIEF-EF domain score were similar across repeated injections. The post-injection EF domain score varied little and actually tended to increase. The time from previous injection to request for a further injection also remained steady throughout the different injections. There was a large decrease in the number of men requesting further injections, especially after the fourth. This is because of the retrospective nature of the study as well as the fact that fewer individuals requested additional injections over time.

The combined pharmacological treatment for ED was unchanged in the vast majority of participants with repeated injections. After the first BTX/A *ic*, only 4/89 participants increased the dose, either of PDE5-Is by combining on-demand PDE-I with daily tadalafil 5 mg or of PGE1 ICI’s. Conversely, 16/89 participants reduced their pharmacological ED treatment, including 1 who switched from PGE1 ICIs to PDE5-Is.

The overall response rate to BTX/A *ic* was 77.5%, including 85.7% in men with mild ED on treatment at baseline (n = 42), 79% in men with moderate ED on treatment at baseline (n = 19) and 64.3% in men with severe ED on treatment at baseline (n = 28). The response rate was similar in men initially treated with PDE5-Is and PGE1 ICI. The response rates to ona/ abo/ incobotulinumtoxinA were, respectively, 79.3, 75 and 78.1% (p = 0.16). Over time, the response rate increased with repeated injections: 67.5%, 87.5% and 94.7% following, respectively, the second (n = 77), third (n = 32) and fourth (n = 19) injections.

**Figure 3:**
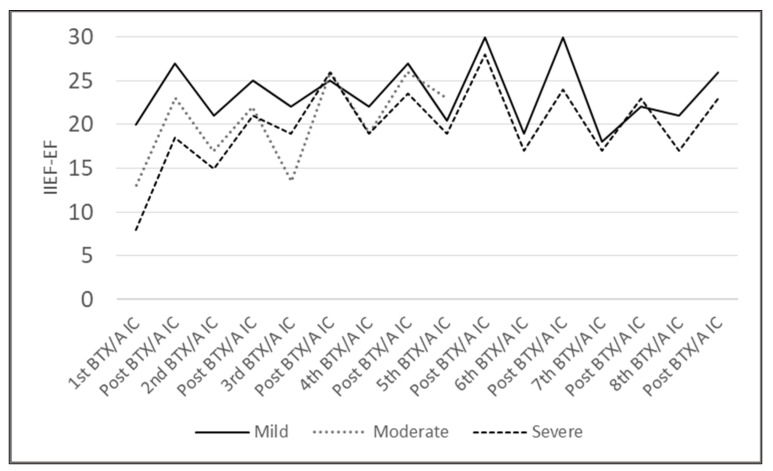
Median Erectile Function domain score of the International Index of Erectile Function (IIEF-EF) at the time of each repeated BoNT/A intracavernosal injection (BTX/A *ic*) during the second month post-injection over time according to the severity of ED on treatment at baseline.

Figure 3 shows the median change in EF domain score across repeated BTX/A *ic* according to the severity of the ED at baseline on treatment. For the seventh and eighth BTX/A *ic*, only one participant had mild ED, and only one had severe ED. 

Four men reported mild or moderate penile pain at the time of injection (1.5% of the total number of injections). One experienced a burn at the penile crus caused by the penile loop. The wound took 10 days to heal and required local care. BTX/A *ic* did not have any impact on the rate of priapism associated with ICIs. No general side effects were reported. 

## 3. Discussion

This case series study of long-term follow-up data on men with ED who were insufficient responders to registered pharmacological treatments and were treated with repeated BTX/A *ic* injections as an add-on therapy is the first to provide preliminary evidence for the sustained long-term effectiveness of repeated BTX/A *ic*. 

The results relating to safety are consistent with those of previous studies [11,12,13,14,15,16] and confirm that BTX/A *ic* is well tolerated and has a consistent safety profile over long-term, repeated treatment with no new safety signal. The only adverse effects were related to the intracavernosal injection procedure itself. This suggests that BTX/A *ic* does not have a cumulative dose or duration toxicity.

Because of the retrospective nature of this study, the number of injections differed for each participant. In addition, the duration of the effectiveness of BTX/A *ic* differed for each participant, with a median duration of around 9 months. Men for whom BTX/A *ic* had a longer duration of effect received fewer treatments, and those with a shorter duration of effect received more treatments during the same study period. Furthermore, the men were treated “as needed” on the basis of their request for a further injection and the fulfillment of prespecified criteria, i.e., ≥5 months since the previous injection and an EF score < 26. This is consistent with standard clinical practice except that BTX/A *ic* was provided free of charge. This is a limitation of the study; paying for the treatment could influence the request for follow-up injections. Furthermore, the BTX/A *ic* outcome should be balanced with the number of men who withdrew from treatment because of an insufficient response. These withdrawals may have biased the increase in response rate over time.

Although the duration of the effect of BTX/A *ic* depended on the individual, the overall median duration of effect of BTX/A *ic* across all participants was similar to the duration reported in studies of intradetrusor BTX/A injection for neurogenic detrusor overactivity [21]. Furthermore, the duration of effect of BTX/A *ic* appeared to be stable across repeated treatments, although the results must be interpreted with caution in view of the limitations relating to the sample size and the number of treatments.

Interestingly, and conversely to previous findings regarding the effectiveness of the first BTX/A *ic* in men with ED and insufficient response to standard pharmacological treatment [16], ED severity on treatment prior to BTX/A *ic* did not seem to influence the response to repeated treatment after the first injection, as shown in Figure 2. This might be explained by the fact that only the best responders requested further treatment among those with severe ED, whereas those with smaller responses did not request further injections.

The present study is largely limited by its retrospective design and by the lack of a control group. Accordingly, unanswered questions remain. Because of the non-placebo-controlled design, the placebo effect in this group of men with difficult-to-treat ED is unknown. However, it is noteworthy that, according to the available randomized placebo-controlled clinical trials in non-responders to PDE5-Is and PGE1 ICI with ED of vascular origin, the placebo effect of BTX/A *ic* is very low [11,12,13]. It is also noteworthy that in our series, the treatment was not individualized. All participants were treated with a fixed dose of BTX/A *ic*. One might speculate that increasing the dose in non-responders to the first BTX/A *ic* might improve the response rate to the second injection.

In conclusion, this study showed that a significant proportion of men with ED and an insufficient response to current pharmacological treatments who experience a clinically meaningful improvement in erectile function after the first BTX/A *ic* treatment continue to experience long-term improvements. The improvement in erectile function after subsequent treatments was consistent and durable with no safety signal. This preliminary evidence suggests that BTX/A *ic* may be an effective long-term add-on therapy for men with difficult-to-treat ED. Large controlled studies are now essential to confirm this finding. 

## 4. Materials and Methods

### 4.1. Participants

We used data from a pilot open-label study that we launched in 2017 to assess the safety and effectiveness of BTX/A *ic* as an add-on therapy for ED. The inclusion criteria were heterosexual men aged ≥18 years who had shown an insufficient response for at least 3 months to the registered PDE5-Is at the highest approved dose, either on-demand or daily (tadalafil), or to PGE1 ICIs with a dose up to 60 µg. Insufficient response to the pharmacological ED treatment was defined as an IIEF-EF domain score < 26 on treatment [20]. BTX/A *ic* was performed as previously described [14]. Briefly, an adjustable penile loop ring was placed at the penile crus for 30 mins. Then, two syringes with a 13 mm long 29 ½ G needle were used to deliver 0.5 ml of BTX/A into each corpus cavernosum. 

For the current study, we included data from all men who had undergone at least two BTX/A *ic* injections. Participants were categorized according to the ED etiology(ies) and/or risk factor(s) and ED severity according to the IIEF-EF score on their current pharmacological treatment prior to BTX/A *ic*. 

### 4.2. BTX/A ic Formulations

We successively tested abo (Dysport^®^)- ona (Botox^®^)- and inco (Xeomin^®^)- botulinumtoxinA at the following doses: 500 U, 100U and 100U, respectively. The results for the safety and effectiveness of a single *ic* injection of these three formulations of BTX/A as an add-on therapy to the current ED pharmacological treatment have been published elsewhere [14,15,16]. Clinical studies in other pathologies have demonstrated that the potency of ona- and inco-botulinumtoxin A is identical. Therefore, they can be compared using a 1:1 conversion ratio [22]. The most commonly reported conversion ratios between onabotulinumtoxinA (or incobotulinumtoxinA) and abobotulinumtoxinA are 1:3 and 1:4 [23]. We found a similar effect on erectile function when PDE5-Is or ICIs were continued after abobotulinumtoxinA (Dysport^®^) 500U, onabotulinumtoxinA (Botox^®^) 100U or incobotulinumtoxinA (Xeomin^®^) 100U single *ic* injection [14,15,16]. Accordingly, for the sake of clarity, the current data are presented without distinction between the different BTX/A formulations. 

### 4.3. Repetition of Injections

After the first BTX/A *ic*, further BTX/A *ic* injections were performed at the individual’s request. The reason given was most often a decrease in effectiveness over time. Some men wished to gain additional improvement in erectile function. We arbitrarily waited at least 5 months before reinjecting. It is important to note that the men received the BTX/A *ic* injections free of charge. After each injection, a first follow-up consultation (most often by telephone) took place during the second month post-injection to assess the safety and the EF domain score and to monitor any change in ED pharmacological treatment. Then, a second follow-up consultation was arranged, usually during the seventh month post-injection or on the individual’s request if the effectiveness had decreased. Throughout the study, apart from their current pharmacological ED treatment, none of the men used any additional non-pharmacological treatments for ED after BTX/A *ic* injection, such as a penile ring or vacuum-device therapy.

### 4.4. Endpoints 

The primary endpoint was the IIEF-EF domain score during the second month post-injection. The secondary endpoints were (1) side effects identified via self-report at the time of injection and during the follow-up and (2) the response to BTX/A *ic* defined as a clinically relevant improvement demonstrated by the achievement of the minimal clinically important difference (MCID) for the EF domain score at the 2-month follow-up visit after each injection. The MCID was adjusted according to the baseline severity of ED (2 points = mild, 5 points = moderate and 7 points = severe) [24] and compared to baseline EF score, i.e., prior to the first injection. Men for whom the MCID was achieved were defined as responders. 

### 4.5. Statistics 

Continuous variables are expressed as means and standard deviations. IIEF-EF domain score, time between two injections and duration of ED are presented as medians (10^th^–90^th^ percentiles) in the box and whisker plot in Figure 2 and first–third quartile in Figure 3. A Kruskal–Wallis test was used to compare non-parametric data. No formal sample size calculation was performed, and all analyses were exploratory. *p* < 0.05 was considered statistically significant. GraphPad Prism v9, San Diego, CA 92108, USA, was used for all analyses.

### 4.6. Ethics

The present database was approved by the French Data Protection Authority (Commission Nationale Informatique et Libertés) according to the national legislation for retrospective studies (registration number 2209010v0). Medical files were anonymized, and participants were informed that they could deny access to their personal and medical data at any time. Written informed consent was obtained from each participant.

## Figures and Tables

**Table 1:** Characteristics of the men who requested at least a second BoNT/A intracavernosal (*ic*) injection. For erectile function (ED) risk factors and etiologies, the total is >100% because several men had more than one risk factor and/or etiology. PDE5-Is: phosphodiesterase type 5 inhibitors; PGE1 ICI: intracavernosal injections of prostaglandin E1.

	Patients Who Requested at Least a Second Injection (n = 92)
Age (years), mean (SD)	52.2 (13)
ED duration (years), median (first quartile–third quartile)	5 (2–10)
IIEF-EF domain score on treatment prior to BTX/A *ic* median (first quartile–third quartile)	14.5 (9–19)
**ED severity according to IIEF-EF domain score on treatment** **prior to BTX/A *ic* [20]**	
Severe n (%)	31 (34)
Moderate n (%)	19 (20)
Mild n (%)	42 (46)
**ED risk factors and/or etiologies**	
Cardiometabolic n (%)	38 (41)
Spinal cord injury n (%)	43 (46)
Post-prostatectomy n (%)	14 (15)
No identified organic risk factor/etiology or other comorbidity(ies) n (%)	15 (16)
**ED treatment prior to BTX/A *ic***	
PDE5-Is n (%)	74 (80)
PGE1 ICIs n (%)	24 (26)
mean dose PGE1 ICI (µg) (SD)	40 (17)
**BTX/A type**	
Onabotulinumtoxin A n (%)	29 (32)
Abobotulinumtoxin A n (%)	28 (30)
Incobotulinumtoxin A n (%)	35 (38)

## Data Availability

The data presented in this study are available in this article.
